# Establishing a Prognostic Signature Based on Epithelial–Mesenchymal Transition-Related Genes for Endometrial Cancer Patients

**DOI:** 10.3389/fimmu.2021.805883

**Published:** 2022-01-14

**Authors:** Jinhui Liu, Guoliang Cui, Shuning Shen, Feng Gao, Hongjun Zhu, Yinghua Xu

**Affiliations:** ^1^ Department of Gynecology, The First Affiliated Hospital of Nanjing Medical University, Nanjing, China; ^2^ Department of Gastroenterology, The Second Affiliated Hospital of Nanjing University of Chinese Medicine, Nanjing, China; ^3^ Department of Orthopedic Surgery, The First Affiliated Hospital of Nanjing Medical University, Nanjing, China; ^4^ Department of Oncology, Nantong Third People’s Hospital Affiliated to Nantong University, Nantong, China; ^5^ Department of Radiation Oncology, Nantong Third People’s Hospital Affiliated to Nantong University, Nantong, China

**Keywords:** epithelial–mesenchymal transition, endometrial cancer, The Cancer Genome Atlas, prognosis, immunity

## Abstract

**Backgrounds:**

Epithelial–mesenchymal transition (EMT) is a sequential process where tumor cells develop from the epithelial state to the mesenchymal state. EMT contributes to various tumor functions including initiation, propagating potential, and resistance to therapy, thus affecting the survival time of patients. The aim of this research is to set up an EMT-related prognostic signature for endometrial cancer (EC).

**Methods:**

EMT-related gene (ERG) expression and clinical data were acquired from The Cancer Genome Atlas (TCGA). The entire set was randomly divided into two sets, one for contributing the risk model (risk score) and the other for validating. Univariate and multivariate Cox proportional hazards regression analyses were applied to the training set to select the prognostic ERGs. The expression of 10 ERGs was confirmed by qRT-PCR in clinical samples. Then, we developed a nomogram predicting 1-/3-/5-year survival possibility combining the risk score and clinical factors. The entire set was stratified into the high- and low-risk groups, which was used to analyze the immune infiltrating, tumorigenesis pathways, and response to drugs.

**Results:**

A total of 220 genes were screened out from 1,316 ERGs for their differential expression in tumor versus normal. Next, 10 genes were found to be associated with overall survival (OS) in EC, and the expression was validated by qRT-PCR using clinical samples, so we constructed a 10-ERG-based risk score to distinguish high-/low-risk patients and a nomogram to predict survival rate. The calibration plots proved the predictive value of our model. Gene Set Enrichment Analysis (GSEA) discovered that in the low-risk group, immune-related pathways were enriched; in the high-risk group, tumorigenesis pathways were enriched. The low-risk group showed more immune activities, higher tumor mutational burden (TMB), and higher CTAL4/PD1 expression, which was in line with a better response to immune checkpoint inhibitors. Nevertheless, response to chemotherapeutic drugs turned out better in the high-risk group. The high-risk group had higher *N* ^6^-methyladenosine (m^6^A) RNA expression, microsatellite instability level, and stemness indices.

**Conclusion:**

We constructed the ERG-related signature model to predict the prognosis of EC patients. What is more, it might offer a reference for predicting individualized response to immune checkpoint inhibitors and chemotherapeutic drugs.

## Introduction

Endometrial cancer (EC) is one of the most common gynecologic cancer, with rising incidence and associated mortality (1). According to the American Cancer Society, the 5-year relative survival (2010–2016) of uterine corpus cancer was 81%; the probability of dying from cancer of uterine corpus was 0.6% (2015–2017). Although the prognosis of EC is better than that of cervical and ovarian cancers, it is meaningful to screen out the high-risk part of EC patients who would have a higher possibility of advanced cancer and early death.

Epithelial–mesenchymal transition (EMT) is a biological process (BP) where epithelial cells gain mesenchymal features. During this process, cells in hybrid EMT state express both epithelial and mesenchymal biomarkers, such as E-cadherin, vimentin, keratin 5, keratin 14, and Cdh2. Through EMT-related pathways, cells gain stem-like features, reduced cell polarity, weakened cell–cell adherence, and the ability to migrate. In cancers, these cells present high metastatic potential (2). Some studies have revealed the role of EMT in EC. EMT status, represented by both reduced E-cadherin and nuclear expression of Snail, was found to be significantly related to clinical features including myometrial invasion, positive cytology, and overall survival (OS) (3), suggesting that EMT signature could be a prognostic factor of EC. As to the molecular mechanism behind, estrogen-related receptor alpha (ERRα) was reported to participate in the TGF-β-induced EMT through cancer–stromal interactions in EC cells (4). Ubiquitin-conjugating enzyme E2C (UBE2C), which is regulated by estrogen, promotes EMT *via* p53 in EC (5). What is more, PD-L1 was found to be modulating EMT and cancer stem cell (CSC)-like phenotype through several signaling pathways (6), which inspired us to investigate the association between EMT status and response to immune checkpoint inhibitor therapy in EC. Tumors are infiltrated by immune cells, such as T cells, NK cells, macrophages, and dendritic cells (DCs), composing the microenvironment around cancer cells. Some inflammatory cells like macrophages shift their ground to support cancer cells during long-term crosstalk with them, which might contribute to resistance to drug therapy. Tumors escape from regular immune recognition by regulating immune checkpoints. The current risk stratification models used in EC mostly stick to clinical features (stage, histological type, and grade), but genomic factors have not been applied to standard clinical use (7). Therefore, we tried to establish an EMT-related gene (ERG)-related risk model for EC in prognosis and might offer a reference for individual treatment in the future.

## Materials and Methods

### Public Data Sources

ERGs were attained from a previous study of EMT-related signatures for CRC (8). ERG expression data and clinical features of 522 EC patients and 23 normal samples were downloaded from The Cancer Genome Atlas (TCGA), and the transcriptome data files were “FPKM”. The clinical and pathological characteristics of the tumor samples are shown in **Table S1** (*p* > 0.05, chi-squared test).

### Identification of Overall Survival-Related Epithelial–Mesenchymal Transition-Related Genes

We analyzed 1,316 ERGs between EC and normal tissues with the “limma” package in R software with a cutoff threshold (the adjusted false discovery rate < 0.05 and absolute |log_2_FC|> 2). The different expression levels of ERGs were visualized by heatmap and volcano plot.

### Construction and Validation of the Epithelial–Mesenchymal Transition-Related Gene Signature Model

We randomly divided the entire set (511 samples) into two sets using the R package “caret.” The training set was used for the construction of the risk model, while the testing set and the entire set were used for validation. Univariate Cox regression analysis was performed in the R package “survival”, and multivariate Cox regression analysis in the “survminer” package to pick out the prognostic ERGs. Then we established the risk score with the following formula: risk score = Σ_i_ multi-Cox-coefficient (ERGi) * expression (ERGi). Survival analysis was used to investigate the relationship between ERG-related risk score and OS. The Kaplan–Meier (K-M) analysis was performed with the “survival” package. Principal component analysis (PCA) was used for dimensionality reduction (9). Time-dependent receiver operating characteristic (ROC) was performed with the “survivalROC” package.

### Construction of the Nomogram

Univariate and multivariate Cox regression analyses were used to investigate whether risk score was an independent prognostic factor in the training, testing, and entire cohorts. We further verified the prognostic value of the risk score stratified by clinicopathological parameters. We constructed the nomogram predicting 1-/3-/5-year survival possibility using the “rms” package. The calibration plots were used to validate the prognostic value of the nomogram.

### Gene Set Enrichment Analysis

Gene Set Enrichment Analysis (GSEA) was used to determine the significantly enriched Kyoto Encyclopedia of Genes and Genomes (KEGG) pathways of ERG mRNA.

GSEA (http://software.broadinstitute.org/gsea/index.jsp) was used to identify BPs that are enriched in the gene rank. Based on a model of the risk score, EC samples in the entire set were divided into high-risk and low-risk groups. Comparing the enrichment of BPs, the underlying biological functions of two groups were identified. The collection of annotated gene sets in the Molecular Signatures Database (MSigDB, http://software.broadinstitute.org/gsea/msigdb/index.jsp) was chosen as the reference gene set in GSEA software. The Nom. *p* < 0.05 was chosen as the cutoff criterion (10). The c2.cp.kegg.v7.4.symbols.gmt was chosen as the reference file.

### Evaluation of Tumor Microenvironment

CIBERSORT tool was used to quantify 22 types of immunocyte fractions based on TCGA RNA-sequencing data (11). ESTIMATE algorithm was used to calculate the immune score, stromal score, ESTIMATE score, and tumor purity based on the expression of immune and stromal cells in the tumor microenvironment (12). Infiltration of immune cells was estimated in several ways including TIMER, CIBERSORT, CIBERSORT-ABS, QUANTISEQ, MCPCOUNTER, XCELL, and EPIC. The activity of immune-related pathways was estimated with single-sample GSEA (ssGSEA) (13).

### Immune Prognostic Signature Analysis

Immune prognostic signature (IPS) can be obtained in an unbiased manner using machine learning method based on four major gene categories (PD1, PD-L1, PD-L2, and CTLA4) that determine immunogenicity. The IPS was calculated using z-scores of representative genes associated with immunogenicity. The IPSs of patients with uterine corpus endometrial carcinoma (UCEC) were extracted from The Cancer Immunome Atlas (TCIA) (https://tcia.at/home) (14).

### Somatic Mutation, Tumor Stemness, and Drug Sensitivity Analysis

The mutation data of endometrial carcinoma patients were obtained from TCGA (Data Category = copy number variation; “maf” file). The top 10 mutation genes were visualized by fall plots using the “maftools” packages in R software (15). In addition, the correlation between tumor mutational burden (TMB) and risk score was also assessed. As previously reported, one-class logistic regression (OCLR) was used to calculate the stemlike indices for each endometrial carcinoma sample (16). The response to chemotherapy and small-molecule drugs in UCEC patients was determined using a public database called Genomics of Drug Sensitivity in Cancer (GDSC; https://www.cancerrxgene.org). The half-maximal inhibitory concentration (IC50) was estimated, which represented the drug response (17). The NCI-60 database is currently the most widely used for cancer drug testing, which was accessed through the CellMiner interface (https://discover.nci.nih.gov/cellminer) (18). Pearson’s correlation analysis was performed to explore the underlying drug sensitivity difference between the high- and low-risk groups.

### Consensus Clustering Analysis

TCGA UCEC cohort was clustered into different groups according to the consensus expression of 10 ERGs with “Consensus Cluster Plus” in R (19). Then we used the K-M method and log-rank test to obtain the OS data between different clusters. A chi-square test was carried out to compare the distribution of age, histologic type, tumor status, stage, and grade between two clusters.

### Quantitative Real-Time PCR

TRIzol reagent (Thermo Fisher Scientific, USA) was used to extract total RNA from 16 EC tissues and 16 normal tissues, and cDNA was reverse-transcribed by Revert Aid First Strand cDNA Synthesis kit (Thermo Fisher Scientific, USA). The qRT-PCR was conducted by SYBR-Green PCR kit (Takara, Tokyo, Japan), and the cycle threshold (CT) of 10 ERGs was recorded. The relative expression of the target gene was estimated using the 2^−ΔΔCT^ method. The primer sequences are listed in **Table S2**.

### Statistical Analysis

All the analyses were performed in the R software (version 4.1.0). Wilcoxon test was used to compare the continuous variables that were not normally distributed. A *p*-value of less than 0.05 was considered statistically significant.

## Results

### Searching for Epithelial–Mesenchymal Transition-Related Genes in Endometrial Cancer

To screen out differently expressed ERGs in EC, we compared the mRNA expression profiles of 552 tumor samples and 23 normal samples from TCGA database. Out of 1,316 ERGs, 220 showed a significant difference, in which 122 were upregulated (log_2_(fold change) > 2), while 98 were downregulated (log_2_(fold change) < −2). The ERGs were displayed with the clustering heatmap and volcano plot (**Supplementary Figures 1A, B** **)**. Gene Ontology (GO) function analysis was divided into three groups: BP group, cellular compartment (CC) group, and molecular function (MF) group. In the BP group, ERGs were mainly involved in the extracellular matrix organization, extracellular structure organization, and external encapsulating structure organization. ERGs in the CC group were mainly enriched in the collagen-containing extracellular matrix, external side of the plasma membrane, and secretory granule lumen. ERGs in the MF group were mainly involved in receptor-ligand activity, signaling receptor activator activity, and cytokine activity (**Supplementary Figure 1C**). The results of KEGG pathway enrichment revealed that the ERGs were mainly concentrated in cytokine–cytokine receptor interaction, microRNAs in cancer, and transcriptional misregulation in cancer (**Supplementary Figure 1D**).

### Identification of Hub Epithelial–Mesenchymal Transition-Related Genes and Development of a Prognostic Index

**Figure 1:**
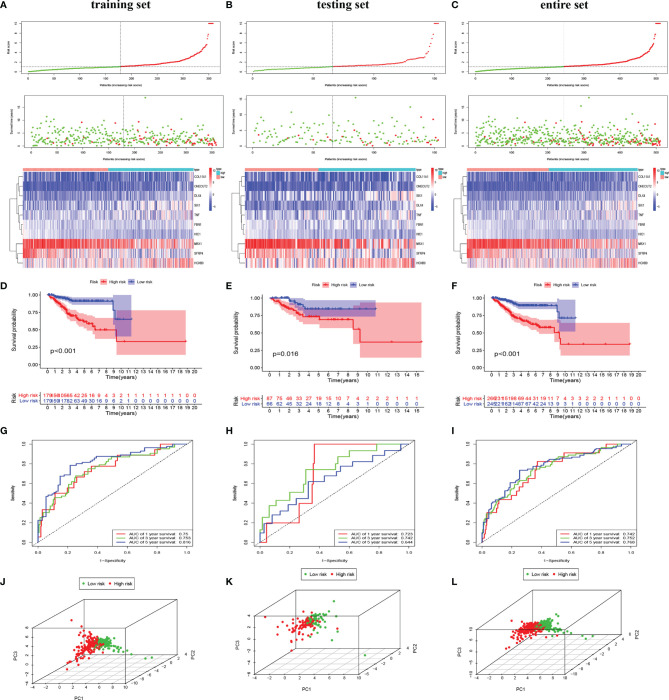
Identification and validation of the EMT-related genes signature in the training, testing, and entire sets. The risk curve and scatter plot of each sample reordered by risk score and the heatmap showed the expression profiles of 10 ERGs in the low-risk group and high-risk group in the training set **(A)**, testing set **(B)**, and entire set **(C)**. Each square represents a clinical sample, and its color is associated with the gene expression. The higher the gene expression, the darker the color (red represents upregulated genes, and blue represents downregulated genes). Kaplan–Meier survival curves and area under the curve (AUC) value of the prognostic factors of 10 ERGs in the training set **(D, G)**, testing set **(E, H)**, and entire set **(F, I)**. **(G)** PCA reveals the difference between the high-risk and low-risk groups in the training set **(J)**, testing set **(K)**, and entire set **(L)**. EMT, epithelial–mesenchymal transition; ERG, EMT-related gene; PCA, principal component analysis.

To investigate the prognostic value of 200 candidate genes associated with EMT, we randomly split the entire set (511 tumor samples) into the training set (n = 153) and the testing set (n = 358). There were no significant differences in clinical factors including age, histological type, grade, and stage between the training set and the testing set by chi-square test and Wilcoxon rank-sum test (**Table S1**). After univariate Cox regression was used to combine clinical information with transcriptional profiles, a total of 31 ERGs associated with survival were identified in the training set (**Table 1**). To generate a prognostic ERG signature model (risk score), multivariate Cox proportional hazards regression analysis was applied to evaluate the connection between ERGs and OS in the training set, and 10 ERGs were identified as the prognostic ERGs (**Table S3**). At last, the ERGs FBN1, HIC1, SFRP4, COL11A1, ONECUT2, HOXB9, DLX4, MSX1, TNF, and SIX1 were included in our prognosis model with the formula of “Risk score = 0.1019 * FBN1 − 0.2529 * HIC1 − 0.0076 * SFRP4 + 0.0544 * COL11A1 + 0.1850 * ONECUT2 + 0.0057 * HOXB9 + 0.1853 * DLX4 − 0.0009 * MSX1 + 0.0265 * TNF + 0.0392 * SIX1 in the training set. In the training set, the median risk score of the training set was the cutoff to divide samples into the high-risk group and low-risk group. To reflect the association between the risk score and prognosis of EC, we dotted the samples of different risk scores according to their survival time. The heatmap showed an expression level of 10 ERGs in the high-risk and low-risk groups (**Figure 1A**). More red dots in the high-risk group indicated that more patients died in less than 5 years, and the K-M analysis suggested that the survival outcome of the low-risk group was significantly better (**Figure 1D**). The ROC curve revealed that our predictive model exhibited good sensitivity and specificity in predicting EC patient OS (5 years, area under the curve (AUC) = 0.816; 3 years, AUC = 0.753; 1 year, AUC = 0.75) (**Figure 1G**). The PCA showed that the samples in the two groups were distributed in different directions (**Figure 1J**).

**Table 1:** Univariate Cox regression analysis of 31 EMT-related genes in EC in the training set.

Genes	HR	Low 95% CI	Up 95% CI	*p*-Value
CTHRC1	1.006	1.001	1.011	0.010
FBN1	1.092	1.022	1.167	0.009
HIC1	0.700	0.511	0.960	0.027
GRIN1	0.519	0.274	0.984	0.045
SFRP4	0.987	0.977	0.998	0.022
COL11A1	1.084	1.043	1.127	<0.001
SOX17	0.995	0.991	0.998	0.005
SZH2	1.071	1.014	1.131	0.013
AURKA	1.053	1.014	1.093	0.007
POSTN	1.017	1.001	1.033	0.042
SPDEF	0.993	0.989	0.998	0.002
IL6	1.016	1.005	1.028	0.006
FOXA2	0.981	0.963	0.999	0.039
ONECUT2	1.253	1.049	1.497	0.013
HOXB9	1.008	1.002	1.013	0.005
APLP1	1.013	1.002	1.025	0.027
DLX4	1.239	1.134	1.354	<0.001
BIRC5	1.022	1.005	1.039	0.012
NTRK3	3.122	1.002	9.732	0.050
FBN2	1.013	1.001	1.026	0.028
CPEB1	1.806	1.066	3.059	0.028
HMGB3	1.018	1.004	1.033	0.012
MSX1	0.998	0.997	0.999	0.002
TIMP2	1.013	1.005	1.021	0.001
PCSK1	1.054	1.023	1.085	<0.001
MMP1	1.016	1.007	1.091	<0.001
TNF	1.033	1.013	1.054	0.001
DLX2	1.048	1.007	1.091	0.023
VCAM1	1.085	1.006	1.171	0.034
TPM1	1.029	1.002	1.058	0.036
SIX1	1.060	1.024	1.098	<0.001

EMT, epithelial–mesenchymal transition; EC, endometrial cancer.

### Validating the Epithelial–Mesenchymal Transition-Related Gene Signature Model in the Testing Set and the Entire Set

To validate the predictive value of the risk score, we performed a similar analysis in the testing set and entire set. In the testing set and entire set, EC samples were stratified into the high-risk and low-risk groups according to the median risk score of the training set. The heatmap showed ERG expression between the high-risk and low-risk groups in the testing set and entire set (**Figures 1B, C** **)**. Survival time plot showed a higher possibility of early death in patients with a higher risk score, and the K-M plot proved that a high-risk score is associated with worse OS (**Figures 1E, F**). Besides, the AUC of the testing set was 0.723 at 1-year survival, 0.742 at 3-year survival, and 0.644 at 5-year survival; the AUC of the entire set was 0.742 at 1-year survival, 0.752 at 3-year survival, and 0.768 at 5-year survival (**Figures 1H, I**). PCA displayed discrete directions of distribution in subgroups (**Figures 1K, L**). All of the above studies demonstrated good performance of our risk score model in the prognosis of EC.

As to the expression and predictive value of each ERG in the risk score formula, we first analyzed the gene expression level in tumor and in normal samples. FBN1, HIC1, and SFRP4 were lower in the tumor than normal tissues; the expressions of other ERGs were the opposite (**Supplementary Figure 2A**). The K-M analysis according to the optimal cutoff expression value of each ERG and the results concluded that 8 ERGs were related to survival possibility (**Supplementary Figure 2B**). Spearman’s correlation analysis showed an interaction among 10 ERGs, and the connection between HIC1 and SFRP4 stood out (**Supplementary Figure 2C**). We further validated the expression level of 10 ERGs using qRT-PCR in clinical sample tissues (**Supplementary Figure 2D**). The results revealed that the mRNA expression levels of DLX4, FBN1, HIC1, HOXB9, ONECUT2, and SIX1 were significantly different between tumor samples and normal tissues, which were consistent with the results from TCGA. However, there was no difference in COL11A1 and TNF expression in clinical sample tissues. In addition, the expression of NSX was significantly lower in EC tissues, which was contrary to the prediction.

### Constructing an Epithelial–Mesenchymal Transition-Related Gene-Featured Predictive Nomogram for Endometrial Cancer

**Figure 2:**
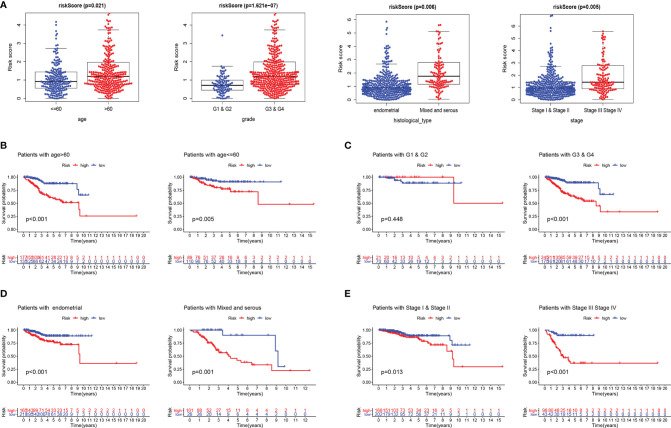
Subgroup analysis of the prognostic value of risk score in EC patients. **(A)** Boxplots of the risk score in EC stratified by age, grade, histological_type, and stage. Prognostic value of risk score in patients with different ages **(B)**, different grades **(C)**, different histological_types **(D)**, and different stages **(E)**. EC, endometrial cancer.

Furthermore, we evaluated the prognostic impact of the risk signature in EC patients with different clinicopathological features in the entire set. Clinical factors including age, histological type, grade, and stage are related to the survival outcome of patients. As shown in **Figure 2A**, risk scores increase with age and disease progression (grade, histological type, and stage). In addition, the risk score reached satisfactory prognostic discrimination in patients with age (**Figure 2B**), grade (**Figure 2C**), histological type (**Figure 2D**), and stage (**Figure 2E**). In aggregate, the above results reveal that the risk model is a promising prognostic classifier for EC patients.

Univariate and multivariate Cox regression analyses were employed to evaluate whether the ERG signature was an independent prognostic indicator for EC patients. The univariate Cox regression analysis proved that risk score, stage, and histological type were independent factors affecting OS in three sets. However, the multivariate Cox regression analysis of the three sets demonstrated only that the signature-based risk score was remarkably correlated with OS (**Table 2**).

**Table 2:** Univariate and multivariable Cox regression analysis of the EMT-related genes and overall survival in different patient sets.

Variable	Univariable model			Multivariable model		
	HR	95% CI	*p*-Value	HR	95% CI	*p*-Value
Training set (n = 153)						
Age	2.024	1.115–3.675	0.021	1.698	0.901–3.199	0.101
histological_type	2.888	1.754–4.755	3.05E−05	1.625	0.929–2.841	0.089
Grade	2.939	1.176–7.342	0.021	1.248	0.461–3.381	0.663
Stage	4.446	2.680–7.374	7.53E−09	3.361	1.914–5.902	2.43E−05
riskScore	1.105	1.075–1.136	1.53E−12	1.084	1.054–1.116	3.34E−08
Testing set (n = 358)						
Age	1.397	0.633–3.086	0.408			
histological_type	3.501	1.609–7.618	0.002	2.903	1.264–6.670	0.012
Grade	5.407	0.732–39.947	0.098			
Stage	3.413	1.575–7.398	0.002	2.251	0.974–5.202	0.058
riskScore	1.036	1.012–1.061	0.003	1.033	1.006–1.061	0.016
Entire set (n = 511)						
Age	1.778	1.112–2.843	0.016	1.498	0.915–2.454	0.108
histological_type	3.044	2.003–4.624	1.84E−07	1.940	1.219–3.087	0.005
Grade	3.363	1.467–7.710	0.004	1.479	0.609–3.592	0.387
Stage	4.116	2.700–6.275	4.82E−11	3.014	1.892–4.799	3.38E−06
riskScore	1.048	1.033–1.062	6.81E−11	1.038	1.021–1.054	5.15E−06

EMT, epithelial–mesenchymal transition.

**Figure 3:**
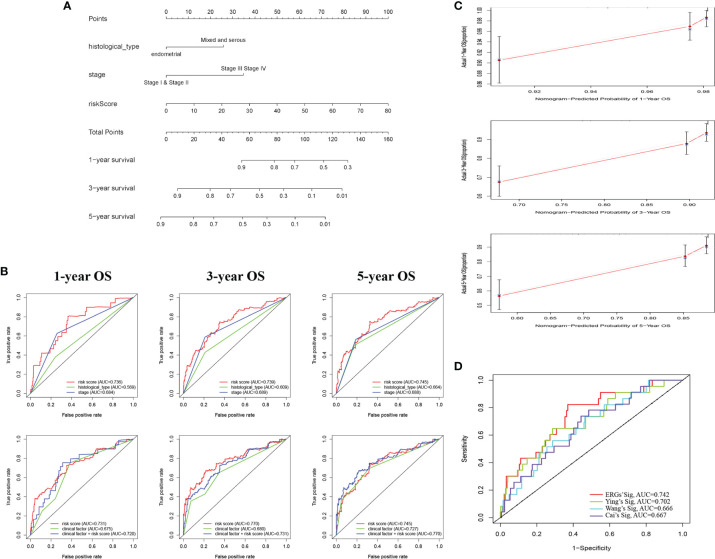
Construction of a nomogram for survival prediction of EC patients. **(A)** The nomogram combining signature with clinicopathological features (histological_type, clinical stage, and risk score). **(B)** ROC curves of 1-, 3-, and 5-year overall survival (OS), indicating that the ERG-based signatures had better predictive ability than other clinical factors. Furthermore, when combined risk score with clinical factors for analysis, the AUC values of 5-year OS increased further, which suggested that the nomograms had superior predictive capacity for the long-term prognosis of EC. **(C)** Calibration plot showing that nomogram-predicted survival probabilities corresponded closely to the actually observed proportions. **(D)** The AUC for ERGs and the existing EMT-related signatures. EC, endometrial cancer; ROC, receiver operating characteristic; EMT, epithelial–mesenchymal transition; ERG, EMT-related gene; AUC, area under the curve.

Based on the ERG-featured risk score and clinical factors, we constructed a nomogram, taking risk score, stage, and histological type into account, to predict the outcome of EC patients (**Figure 3A**). According to the point of three factors, we could calculate the total points so as to predict the 1-/3-/5-year survival possibility of a certain patient. Higher total points mean poorer outcomes. The multi-ROC curves proved that synthesizing clinical factors and risk scores would be better than a single factor (**Figure 3B**). The correlation of ERG expression and risk score with clinical factors are exhibited in **Table S4**. The calibration plots demonstrate the concordance of our nomogram result with the actual circumstances (**Figure 3C**). In order to further prove the predictive performance of the ROC curve for ERGs in this model, we compare three recently published articles on the signatures of the prognostic model in EC (20–22). Based on the same TCGA patient cohort, we found that in this model, the AUC of OS for our signatures is 0.742, which is significantly higher than that of other existing EMT-related signatures (**Figure 3D**).

### Exploring Biological Functions of Epithelial–Mesenchymal Transition-Related Genes

To further investigate the molecular mechanism of these ERGs, we analyzed the expression profile of transcription factors (TFs) between EC and normal endometrium and screened out 27 differentially expressed TFs (**Table S5**), which are shown with the clustering heatmap and the volcano plot (**Supplementary Figures 3A, B** **)**. A regulatory network was also constructed by ERGs with relevant TFs (**Supplementary Figure 3C**). Second, we used GSEA to discover significant pathways of ERGs. The top 5 KEGG pathways enriched in the high-risk group (cell cycle, DNA replication, homologous recombination, EC, and pathways in cancer) were associated with tumorigenesis; those of the low-risk group (asthma, autoimmune thyroid disease, cytokine–cytokine receptor interaction, graft-versus-host disease, and intestinal immune network for IgA production) were immune-related (**Supplementary Figure 3D**). The results indicated that low-risk scores were associated with immune signaling pathways.

### Evaluating Immune Status in Groups Stratified by Risk

**Figure 4:**
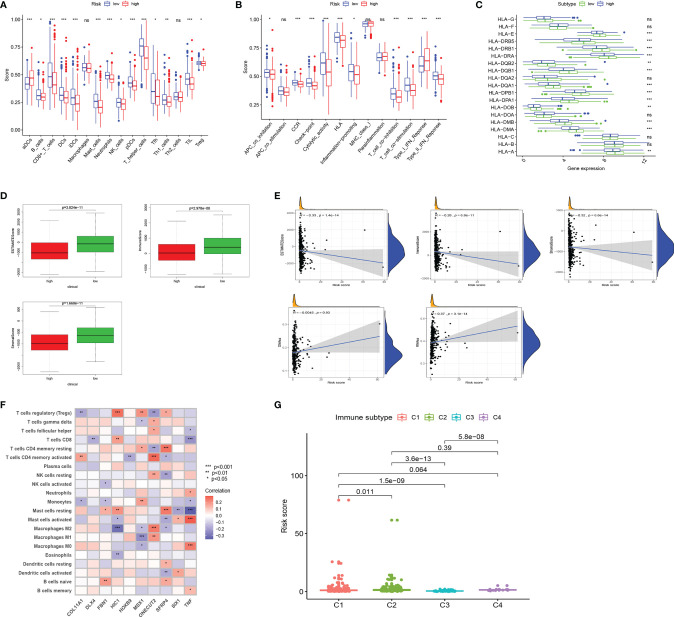
Landscape of immune cell infiltration. **(A)** Comparison of 16 tumor immune-related cells in the high-risk and low-risk groups. **(B)** Comparison of 13 immune-related functions in the high-risk and low-risk groups. **(C)** The distribution of HLA-related genes of the high-risk and low-risk groups. **(D)** Comparison of ESTIMATES score, Immune score, and Stromal score in high-risk and low-risk groups. **(E)** The relationship between risk score and ESTIMATES score, Immune score, Stromal score, RNAss, and DNAss. **(F)** The correlation between 10 ERGs and immune cells infiltration. **(G)** Comparison of the risk score in different immune infiltration subtypes. EC, endometrial cancer; ROC, receiver operating characteristic; EMT, epithelial–mesenchymal transition; ERG, EMT-related gene; AUC, area under the curve; HLA, human leukocyte antigen. Adjusted p-values were shown as ns, not significant; *p < 0.05; **p < 0.01; ***p < 0.001.

Tumor immune-related cells were compared in different risk groups, and ssGSEA showed more immune activities in the low-risk group. The results exhibited that the abundances of activated DCs (aDCs) were significantly decreased in the low-risk group, while in the high-risk group, B cells, CD8+ T cells, DCs, interdigitating DCs (iDCs), mast cells, neutrophils, natural killer (NK) cells, plasmacytoid DCs (pDCs), T helper cells, Tfh, Th1 cells, TIL, and regulatory T cells (Tregs) were markedly decreased (**Figure 4A**). Comparisons of 13 immune-related functions in the high-risk and low-risk groups confirmed the difference of antigen-presenting cell (APC) co-inhibition, CC chemokine receptor (CCR), checkpoint, cytolytic activity, human leukocyte antigen (HLA), inflammation promoting, T-cell co-inhibition, T-cell co-stimulation, type I IFN response, and type II IFN response (**Figure 4B**). We further investigated the expression of key immunity genes, HLA genes, which were mostly higher in the low-risk group (**Figure 4C**). In addition, we calculated ESTIMATE SCORE and found that the low-risk group had a higher ESTIMATE score, immune score, and stromal score (**Figure 4D**). The correlation analysis revealed that risk score was significantly negatively associated with ESTIMATE score, immune score, and stromal score. RNA stemness score (RNAss) and DNA stemness score (DNAss) were performed to measure tumor stemness based on mRNA expression and DNA methylation pattern, respectively. The results indicated that the risk score was not significantly associated with DNAss, but significantly positively correlated with RNAss (**Figure 4E**). We further investigated the association between the expression of 10 ERGs and the tumor-infiltrating immune cells (TIICs) in EC using the TIMER database (**Figure 4F**). To explore the relationship between risk score and immune component, we identified the correlation between risk score and immune infiltration. There are six different types of immune infiltrates in human tumors, corresponding to tumor promotion and tumor suppression, namely, C1(wound healing), C2 (INF-g dominant), C3 (inflammatory), and C4 (lymphocyte depleted) (23). The results showed that a higher risk score was significantly associated with C1, while a lower risk score was significantly associated with C4 (**Figure 4G**).

**Figure 5:**
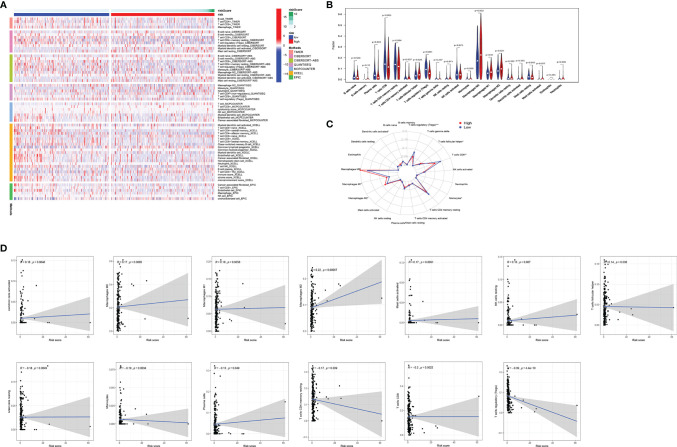
Comparison of immune signature between the high-risk and low-risk groups of EC patients. **(A)** The infiltration of 21 types of immune cells in high-risk and low-risk groups was estimated by TIMER, CIBERSORT, CIBERSORT-ABS, QUANTISEQ, MCPCOUNTER, XCELL, and EPIC database. **(B, C)** Comparison of tumor-infiltrating immune cells between different risk groups. **(D)** The correlation of risk score and tumor-infiltrating immune cells. EC, endometrial cancer.

TIICs contribute to building the microenvironment of tumors. We used TIMER, CIBERSORT, CIBERSORT-ABS, QUANTISEQ, MCPCOUNTER, XCELL, and EPIC to estimate infiltration of 21 types of immune cells in the high-risk and low-risk groups (**Figure 5A**). CD8 T cells, Tregs, and plasma cells have a bigger fraction in the low-risk group; M0/M1/M2 macrophages and T follicular helper cells have a larger proportion in the high-risk group (**Figures 5B, C** **)**. The correlation of risk score and TIICs is shown in **Figure 5D**, which demonstrated that aDCs, macrophages M0, macrophages M1, macrophages M2, mast cells activated, NK cells resting, and T cells follicular helper were positively correlated with a risk score, while risk score was negatively associated with mast cell resting, monocytes, plasma cells, T cells CD4 memory resting, T cells CD8, and Tregs.

### The Prognostic Value of Immune Checkpoint Modulators and Response to Immune Checkpoint Inhibitor

**Figure 6:**
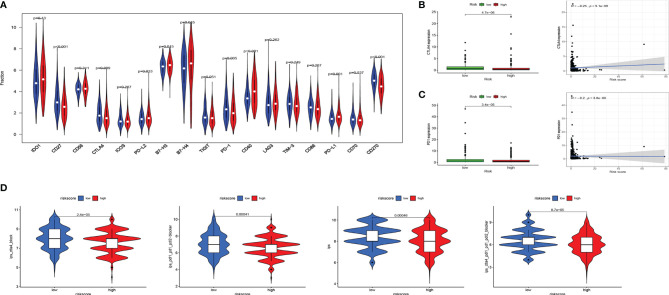
Associations of immune cell infiltration, immune checkpoints expression, and IPS and the EMT-related risk signature. **(A)** The distribution of immunomodulators in high-risk and low-risk groups. **(B, C)** The expression of 2 immune checkpoint molecules (CTLA4 and PD1) in high-risk and low-risk groups and Pearson’s correlation coefficient calculated between risk score and 2 immune checkpoint molecules. **(D)** The association between IPS and the risk score in EC patients. IPS, immune prognostic signature; EMT, epithelial–mesenchymal transition; EC, endometrial cancer.

Immune checkpoint modulators play a critical role in immune cells’ battle with cancer cells. We analyzed the distribution of 17 pivotal modulators in the high- and low-risk groups in the entire set. As a result, 9 of them (CD27, CTLA4, PD-L2, B7-H3, B7-H4, PD-1, CD40, PD-L1, and CD270) have a statistically significant difference in two different risk groups (**Figure 6A**). We focused on CTLA4 and PD1, and the box plot and correlation plot of the two molecules with risk scores demonstrated that patients with low risk have higher CTLA4 and PD1 expression (**Figures 6B, C** **)**. These two molecules might be protective factors in EC.

Then we used Immunophenoscore to estimate the response to immune checkpoint inhibitors (CTLA4-blocker, CTLA4-PD1-PDL1-PDL2-blocker, and PD1-PDL1-PDL2-blocker) in subgroups stratified by risk score. As is shown in **Figure 6D**, the low-risk group had higher IPS, indicating higher immunogenicity of tumors.

### Tumorigenesis Biological Processes and Epithelial–Mesenchymal Transition-Related Gene-Based Risk Score

**Figure 7:**
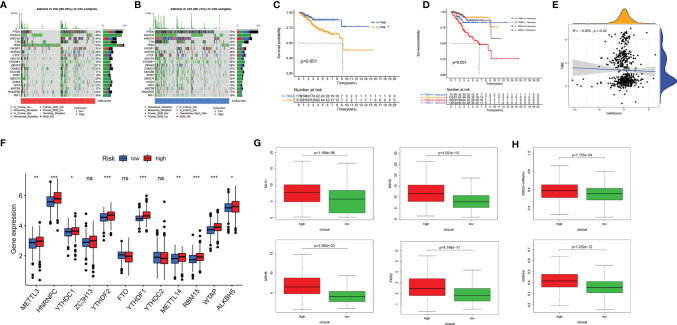
Tumor mutational burden (TMB), somatic mutation, *N* ^6^-methyladenosine, and microsatellite instability (MSI) analysis. Oncoplot displaying the somatic landscape of EC with high-risk group **(A)** and low-risk group **(B)**; the genes are sorted according to their mutation frequency. **(C)** Survival analysis between high-TMB and low-TMB EC patients. **(D)** Two-factor survival analyses of risk score and TMB level. **(E)** Relationship between TMB and EMT-related risk score. **(F)** The expression level of main genes of m^6^A writers, erasers, and readers (HNRNPC, YTHDF1, YTHDF2, RBM15, and WTAP) in high-risk and low-risk groups. **(G)** The expression of MLH1, MSH2, MSH6, and PMS2 in high-risk and low-risk groups. **(H)** mRNA stemness indices between high-risk and low-risk groups. EC, endometrial cancer. Adjusted p-values were shown as ns, not significant; *p < 0.05; **p < 0.01; ***p < 0.001.

Tumorigenesis pathways were enriched in the high-risk group. Therefore, we looked into TMB, *N* ^6^-methyladenosine (m^6^A) RNA modification, microsatellite instability (MSI), and CSC characteristics. Gene mutations lead to the generation and development of tumors. TMB corresponds with the objective response rate for anti-PD-1 therapy across multiple cancer types (24). The oncoplot displays that in the high-risk group, genes of the top 3 mutation rate were tp53, PTEN, and PIK3CA; in the low-risk group, the top 3 mutated genes were PTEN, ARID1A, and PIK3CA. What is more, the mutation frequency of PTEN and PIK3CA was higher in the low-risk group than in the high-risk group (**Figures 7A, B** **)**. The K-M plot shows the relationship of higher TMB and better OS (**Figure 7C**), which was consistent with a better response to immune checkpoint inhibitors in the low-risk group mentioned above. Using both TMB and ERG risk scores could better predict the survival time of EC patients (**Figure 7D**). The was no significant correlation between risk score and TMB (**Figure 7E**).

m^6^A RNA modification regulates the generation and maintenance of CSCs, which drives the progression of cancer (25). We studied the expression level of the main genes of m6A writers, erasers, and readers in the EC cohort. The expression of HNRNPC, YTHDF1, YTHDF2, RBM15, and WTAP was significantly higher in the high-risk group (**Figure 7F**). Approximately 30% of primary ECs are MSI-high/hypermutated (MSI-H), and 13% to 30% of recurrent ECs are MSI-H or mismatch repair deficient (dMMR) (26). MLH1, MSH2, MSH6, and PMS2 were lost more frequently in the high-risk group (**Figure 7G**). Two stemness indices, mRNAsi and EREG-mRNAsi, were utilized to investigate the expression of stem cell-related genes in EC. In the high-risk group, both indices were higher. Oncogenic dedifferentiation marked by CSC characteristics might be another predictive marker of EC (**Figure 7H**).

### Response to Chemotherapeutic Drugs and Epithelial–Mesenchymal Transition-Related Gene-Based Risk Score

**Figure 8:**
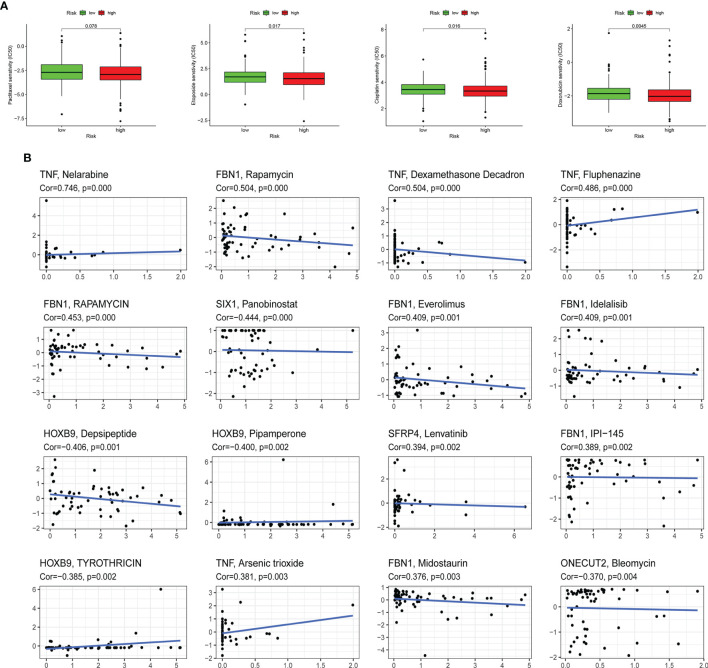
Association between the risk score, ERGs, and chemosensitivity in EC. **(A)** The box plots of the estimated IC50 for paclitaxel, etoposide, cisplatin, and doxorubicin in high-risk and low-risk groups. **(B)** Scatter plot of the relationship between the expression of ERGs and drug sensitivity. EMT, epithelial–mesenchymal transition; ERG, EMT-related gene; EC, endometrial cancer.

The sensitivity of four kinds of chemotherapeutic drugs including cisplatin, doxorubicin, etoposide, and paclitaxel was analyzed (**Figure 8A**). The low-risk group had a higher IC50 of etoposide, cisplatin, and doxorubicin than the high-risk group, which demonstrated that patients with higher risk scores were more sensitive to those chemotherapeutic drugs. In addition, the expression of 10 ERGs in NCI-60 cell lines was investigated, and the relationship between their expression levels and drug sensitivity was revealed at the same time. The results showed that 6 ERGs were correlative to some chemotherapy drug sensitivity (*p* < 0.01, **Figure 8B**).

### Consensus Clustering Analysis of Epithelial–Mesenchymal Transition-Related Genes

By consensus unsupervised clustering of 511 samples from EC patients, we found that 2 clusters had lower values of ambiguously clustered pairs (PAC), which reflected the near-perfect stability of the samples under the correct K value distribution (**Supplementary Figures 4A, B** **)**. We classified the samples into two clusters (k = 2) based on ERG expression (**Supplementary Figures 4C, D** **)**. The clinical characters distributed in two clusters are shown in **Supplementary Figures 4E–H**. The heatmap demonstrates the distinction of ERG expression between two clusters (**Supplementary Figure 4I**). Cluster2 had obviously more MSX1 expression and longer OS (**Supplementary Figure 4J**). The analysis also implied that the genes involved in the signature could have great possibilities to become biomarkers for EC and proved that the signature might have a vital role in the clinical contributions.

## Discussion

As the incidence rate of EC increases and the type of cases becomes complicated, TCGA reported a classification for EC—POLE-mutated, MSI-H, copy number-low, and copy number-high (27)—inspiring us that genomic characters can be a guide for prognoses and treatments. However, some patients still cannot benefit from therapy according to the current classification. Better surveillance and therapeutic regimens are an urgent need. Therefore, we developed an ERG-related signature model as a novel classification tool for EC patients.

EMT has been noticed as a characteristic of tumor cells acquiring both epithelial and mesenchymal features, which is associated with progress and metastasis. In this study, we collected clinical data and expression files of ERGs from TCGA database. From 1,316 ERGs, we found 220 genes expressed differently between tumor and normal samples and finally identified 10 genes (FBN1, HIC1, SFRP4, COL11A1, ONECUT2, HOXB9, DLX4, MSX1, TNF, and SIX1) associated with the prognosis of EC to establish a predicting model. The AUC value based on the training set and the entire set was all above 0.7. Next, taking traditional clinical categories into consideration, we generated an ERG-based predictive nomogram. The nomogram can offer a total score for a specific patient, and the score can identify the predicting 1-/3-/5-year survival possibility.

Among the critical 10 genes, FBN1 encodes fibrillin-1, composed of microfibrils in the extracellular matrix. The mutation in FBN1 was responsible for Marfan’s syndrome and other disorders of connective tissues (28). In EC, FBN1 was identified as a substrate for FBXO2-mediated ubiquitin-dependent degradation. FBOX2 promotes EC proliferation by regulating the cell cycle and the autophagy signaling pathway, whose function would be blocked by the absence of FBN1 (29). HIC1 (Hypermethylated in Cancer1) is a tumor suppressor gene inactivated by epigenetic silence (30). HIC1 is found in a CpG island of chromosome 17p13.3 region, frequently hypermethylated in various types of tumors, and is associated with poorer survival (31). Its methylation density increases from normal tissues to precancerous lesions to cancer. Few papers mentioned the role of HIC1 in EC, except one that found that HIC1 expression significantly reduced in RT-PCR analysis in rat EC cells compared with non-malignant samples, but could not see the same decrease in protein level (32). Our study discovered the association of HIC1 expression level with the prognosis of EC. SFRP4 (secreted frizzled related protein 4) is an extracellular antagonist of the Wnt/β-catenin pathway. Its loss was noticed in aggressive ovarian cancer types and recombinant SFRP4 (rSFRP4) treatment of serous ovarian cancer cells that result in the inactivation of the Wnt/β-catenin pathway, mesenchymal-to-epithelial transition, and decreasing ability to migrate (33). SFRP4 is more frequently downregulated in MSI type of EC compared with microsatellite stable ones (MSS) (34). COL11A1 encodes one of the two alpha chains of type XI collagen. In ovarian cancer, COL11A1 is expressed in the intra/peri-tumoral stromal cells and rare foci of tumor epithelial cells, indicating COL11A1 as a marker of carcinoma-associated fibroblasts and possibly cancer cells undergoing EMT (35). ONECUT2 encodes a member of the one cut family of TFs. It is involved in EMT, resulting in cell growth and invasion in gastric (36) and colorectal and ovarian cancers (37, 38). HOXB9, a TF induces angiogenesis, increased cell motility, and acquisition of mesenchymal characters, thus contributing to lung metastasis of breast cancer (39). HOXB9 induces EMT through TGF-β1-Smad signaling in HCC, promoting migration and invasion of HCC cells (40). DLX4, widely expressed in different types of cancer but absent in most normal adult tissues, induces EMT through directly binding to regulatory regions of TWIST gene (41). What is more, DLX4-mediated EMT in trophoblasts may be a possible pathophysiological mechanism for preeclampsia (42). MSX1 is significantly upregulated in EC, which plays a crucial role in progestin resistance. Knockdown of MSX1 inhibited EMT and improved the therapeutic effect of progesterone (43). TNF-α, a pro-inflammatory cytokine, enhances TGF-β-induced EMT by activating the Smad2/3 signal (44). A similar mechanism is found for SIX1 in papillary thyroid carcinoma (45). Further, SIX1 increases CSC recruit macrophages and stimulate angiogenesis, contributing to the progression of cancer. All the above elucidate the function of these 10 genes in EMT. Our study propels knowledge of the relationship of these genes with the prognosis of EC.

Then, through GSEA, we investigated biological functions of the 10 ERG and we found that our signature was significantly associated with the immuno-microenvironment of the EC. According to the results that immune-related pathways were enriched in the low-risk group, we analyzed immune cell infiltration in two groups. In addition, CD8+ T cells, Tregs, and plasma cells were distributed more in the low-risk group, while macrophages and T follicular helper cells were distributed more in the high-risk group. Previous studies demonstrated that CD8+ count could be an independent prognostic factor of EC (46, 47). In our signature, we found that CD8+ T cells were lower in the high-risk group, leading to a poor prognosis. dMMR was observed to be related to positive PD-L1 expression and high CD8+ cell count (48, 49), in which the subgroup might benefit from immunotherapy. From our study, we found higher CTLA4 and PD1 expressed in the low-risk group and higher IPS, indicating better response to immune checkpoint inhibitors. dMMR was observed more frequently in the high-risk group. Apart from immunotherapy, we also evaluated the effect of chemotherapeutic drugs, including cisplatin, doxorubicin, etoposide, and paclitaxel. Similarly, patients in the low-risk group were more sensitive to chemotherapy. In recent years, in-depth studies have been conducted on ERGs for different immune states of tumors, and a large number of diagnostic and prognostic assessment methods have been identified (20–22, 50). Compared with existing prognostic features, our new prognostic model has greater clinical application potential. Therefore, the signature we established may be helpful to classify patients into different risk groups and offer different recommendations about treatments.

Nowadays, complex predicting models consisting of more cancer-related genes and clinical characters may be a trend for comprehensive individualized diagnosis and treatment. We generated a nomogram taking 10 ERG and clinical features together to identify the 1-/3-/5-year survival possibility of EC patients. However, there are still some restrictions in our study. First, data were based on TCGA database, and validation was performed inside the entire cohort. Second, the mechanism of how these ten genes regulated EMT in EC needs further study. Third, more clinical information about the accuracy of our signature in predicting drug response needs to be collected worldwide.

## Conclusions

In summary, we developed a signature model based on 10 ERG for EC patients and verified its independent prognostic value. What is more, it might offer a reference for predicting individualized response to immune checkpoint inhibitors and chemotherapeutic drugs.

## Data Availability Statement

The datasets presented in this study can be found in online repositories. The names of the repository/repositories and accession number(s) can be found in the article/**Supplementary Material**.

## Ethics Statement

The studies involving human participants were reviewed and approved by The Third People’s Hospital of Nantong. The patients/participants provided their written informed consent to participate in this study.

## Author Contributions

HZ and YX conceived the study and participated in the study design, performance, and manuscript writing. JL, GC, FG, and SS conducted the bioinformatics analysis. All authors read and approved the final manuscript.

## Conflict of Interest

The authors declare that the research was conducted in the absence of any commercial or financial relationships that could be construed as a potential conflict of interest.

## Publisher’s Note

All claims expressed in this article are solely those of the authors and do not necessarily represent those of their affiliated organizations, or those of the publisher, the editors and the reviewers. Any product that may be evaluated in this article, or claim that may be made by its manufacturer, is not guaranteed or endorsed by the publisher.

## Supplementary Material

Supplementary Figure 1 Identification of EMT-related genes (ERGs) from endometrial cancer samples. Heatmap **(A)** and volcano plot **(B)** of ERGs between EC and normal endometrium. Each square represents a clinical sample, and its color is associated with the gene expression. The higher the gene expression, the darker the color. Red dots represent up-regulated ERGs, green dots represent down-regulated ERGs and black dots represented no ERGs. Results of Gene ontology analysis **(C)**, Kyoto Encyclopedia of Genes and Genomes pathways pathway enrichment analysis **(D)**.

Supplementary Figure 2 The expression levels and prognostic value of ERGs. **(A)** Compared with the normal tissues, the expression levels of 10 ERGs in EC. **(B)** Kaplan-Meier survival curves of the high and low expression of ERGs in EC were compared based on the optimal cut-off expression value of each ERG. **(C)** The interaction between 10 ERGs was analyzed by the Spearman’s correlation analysis. **(D)** Expression level of COL11A1, DLX4, PBN1, HIC1, HOXB9, MSX1, ONECUT2, SFRP4, SIX1 and TNF in clinical samples.

Supplementary Figure 3 Identification of survival associated EMT-related genes (ERGs) and relevant transcription factors (TFs). **(A)** Heatmap and **(B)** volcano plot of differentially expressed TFs between EC and normal endometrium. Red dots represented up-regulated TFs, green dots represented down-regulated TFs and black dots represented no TFs. **(C)** Differentially expressed ERGs and regulatory network with relevant TFs. **(D)** Five representative KEGG pathways for the high-risk and low-risk group.

Supplementary Figure 4 Consensus clustering analysis of ERGs. **(A)** cumulative distribution function (CDF) curve of K = 2–10. **(B)** The relative change in area under the CDF curve of K = 2–10. **(C)** Sample clustering heatmap. **(D)** Tracking plot of k in the consensus cluster of EMT-related genes. **(E–H)** The proportion of clinical characters (age, grade, histological type and stage) in two clusters. **(I)** The heatmap of ERG expression in two clusters. **(J)** Prognostic prediction in two clusters by Kaplan-Meier.
