# Supplementary figures and images for: Establishing a Prognostic Signature Based on Epithelial–Mesenchymal Transition-Related Genes for Endometrial Cancer Patients
Source: Front Immunol. 2022 Jan 14;12:805883. doi: 10.3389/fimmu.2021.805883 (PMC8795518; doi:10.3389/fimmu.2021.805883)

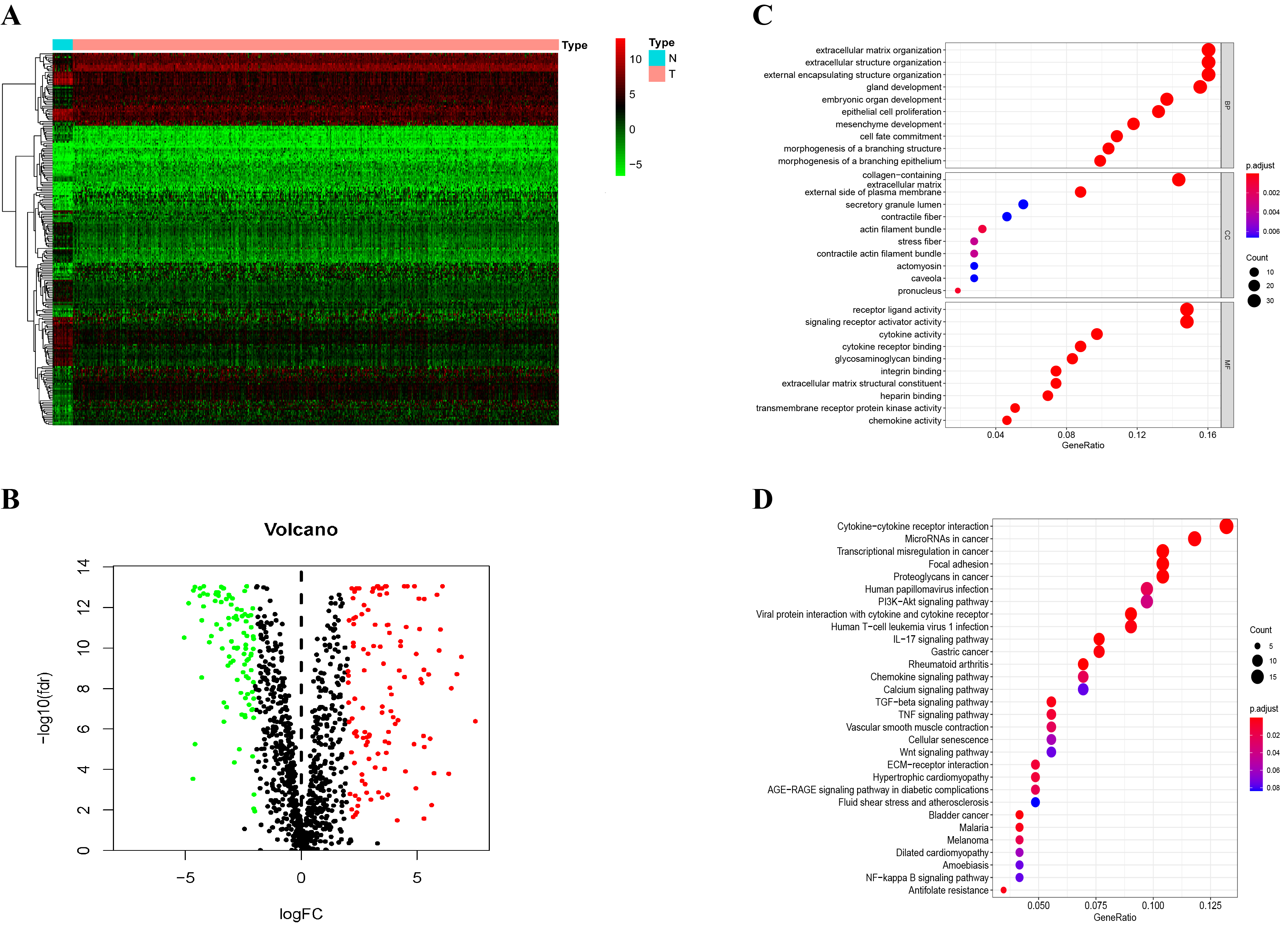

Supplement: Supplementary Figure 1 — Identification of EMT-related genes (ERGs) from endometrial cancer samples. Heatmap (A) and volcano plot (B) of ERGs between EC and normal endometrium. Each square represents a clinical sample, and its color is associated with the gene expression. The higher the gene expression, the darker the color. Red dots represent up-regulated ERGs, green dots represent down-regulated ERGs and black dots represented no ERGs. Results of Gene ontology analysis (C), Kyoto Encyclopedia of Genes and Genomes pathways pathway enrichment analysis (D). [file Image_1.tif]

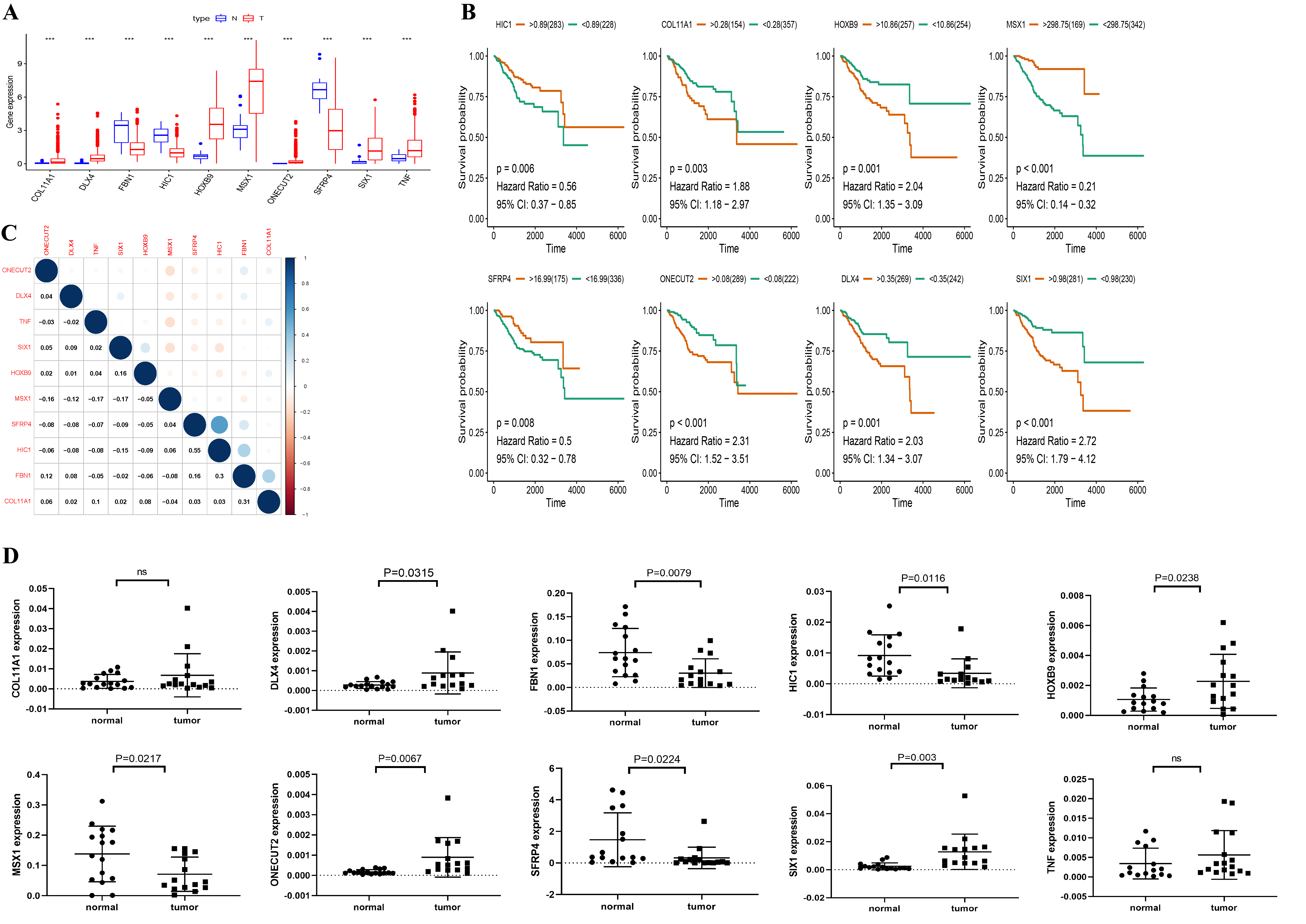

Supplement: Supplementary Figure 2 — The expression levels and prognostic value of ERGs. (A) Compared with the normal tissues, the expression levels of 10 ERGs in EC. (B) Kaplan-Meier survival curves of the high and low expression of ERGs in EC were compared based on the optimal cut-off expression value of each ERG. (C) The interaction between 10 ERGs was analyzed by the Spearman’s correlation analysis. (D) Expression level of COL11A1, DLX4, PBN1, HIC1, HOXB9, MSX1, ONECUT2, SFRP4, SIX1 and TNF in clinical samples. [file Image_2.tif]

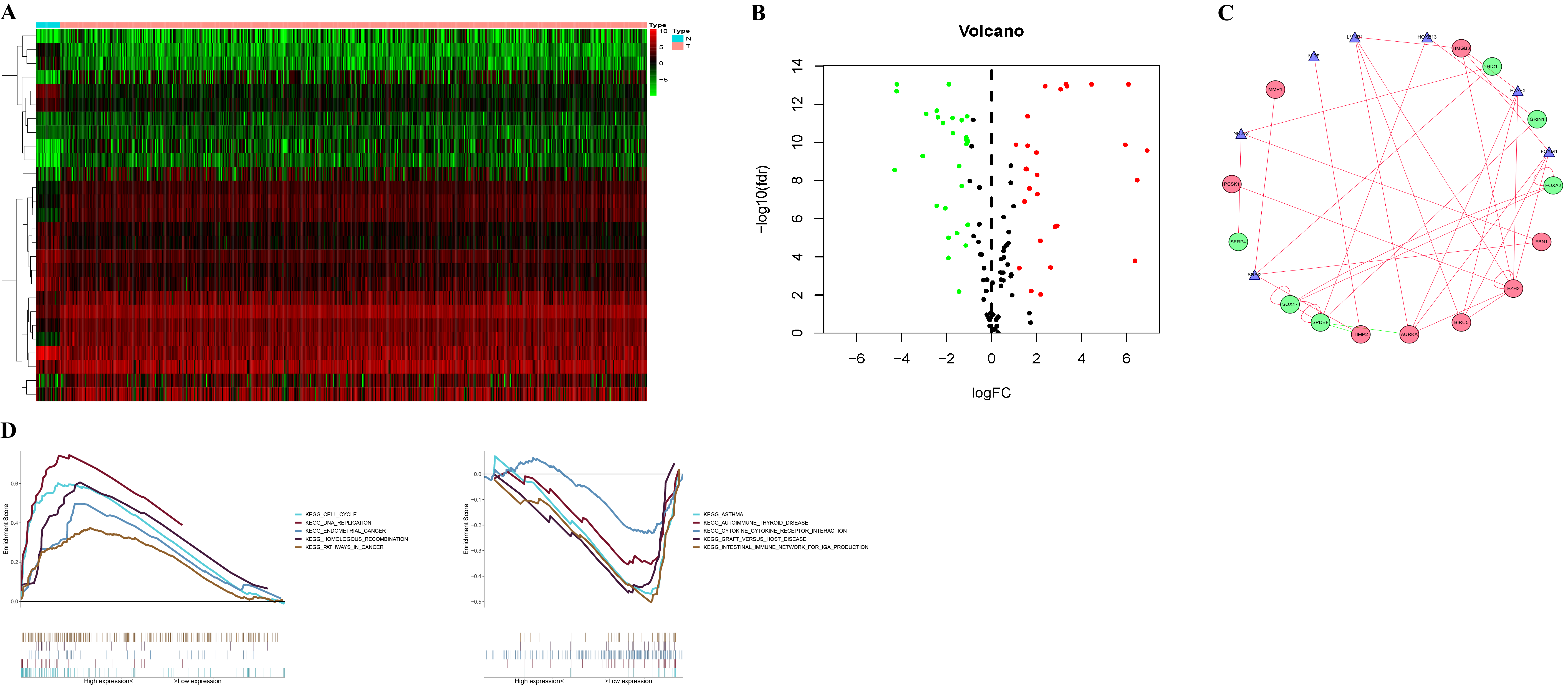

Supplement: Supplementary Figure 3 — Identification of survival associated EMT-related genes (ERGs) and relevant transcription factors (TFs). (A) Heatmap and (B) volcano plot of differentially expressed TFs between EC and normal endometrium. Red dots represented up-regulated TFs, green dots represented down-regulated TFs and black dots represented no TFs. (C) Differentially expressed ERGs and regulatory network with relevant TFs. (D) Five representative KEGG pathways for the high-risk and low-risk group. [file Image_3.tif]

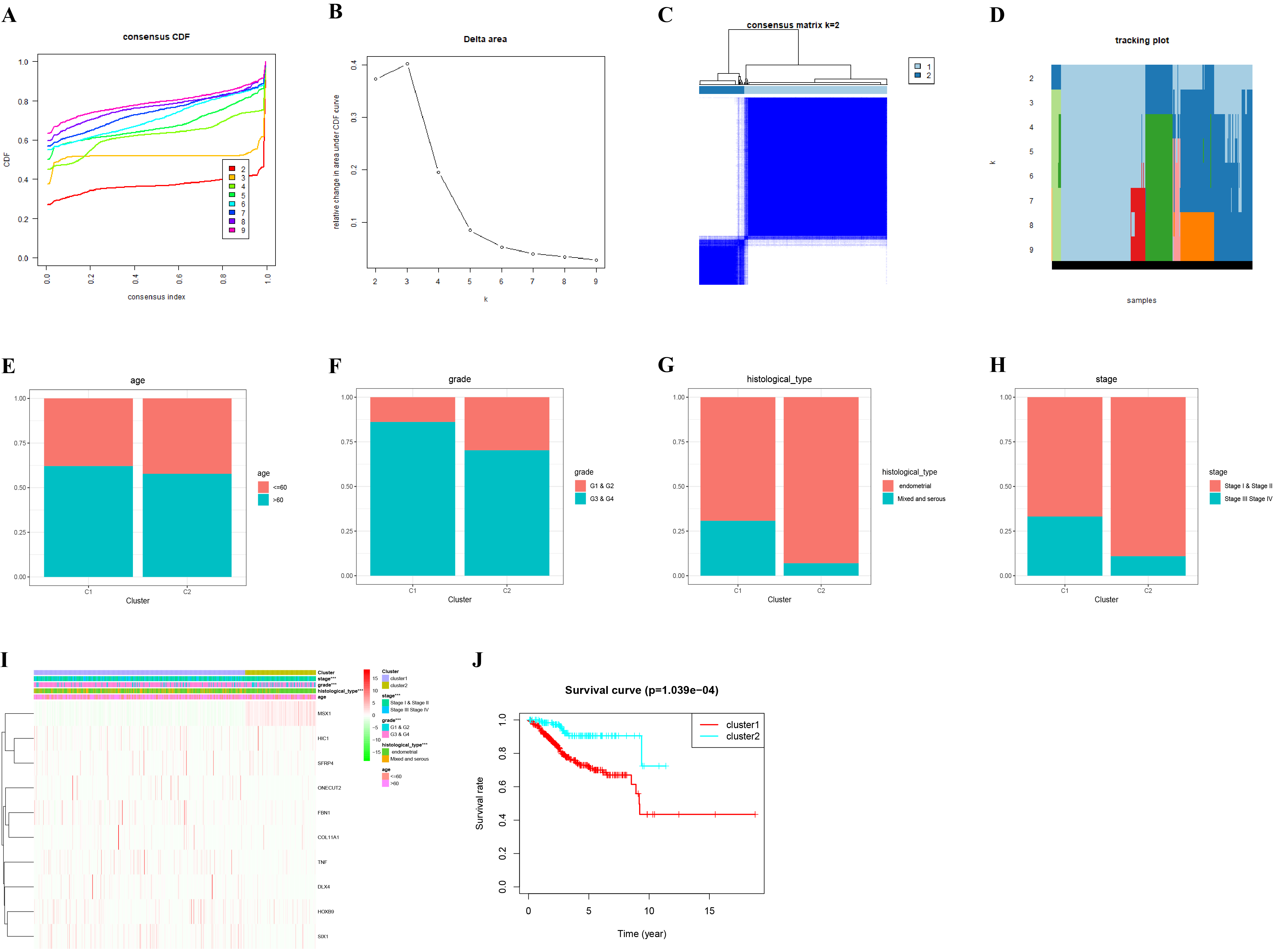

Supplement: Supplementary Figure 4 — Consensus clustering analysis of ERGs. (A) cumulative distribution function (CDF) curve of K = 2–10. (B) The relative change in area under the CDF curve of K = 2–10. (C) Sample clustering heatmap. (D) Tracking plot of k in the consensus cluster of EMT-related genes. (E–H) The proportion of clinical characters (age, grade, histological type and stage) in two clusters. (I) The heatmap of ERG expression in two clusters. (J) Prognostic prediction in two clusters by Kaplan-Meier. [file Image_4.tif]
