# Supplementary material for: Establishing a Prognostic Signature Based on Epithelial–Mesenchymal Transition-Related Genes for Endometrial Cancer Patients
Source: Front Immunol. 2022 Jan 14;12:805883. doi: 10.3389/fimmu.2021.805883 (PMC8795518; doi:10.3389/fimmu.2021.805883)
Supplement: Supplementary file 6 [file Table_1.docx]

**Table S1 Clinical features of EC patients**

| Covariates | Type | Entire set | Testing set | Training set | Pvalue |
| --- | --- | --- | --- | --- | --- |
| age | <=60 | 199(38.94%) | 65(42.48%) | 134(37.43%) | 0.3301 |
| age | >60 | 312(61.06%) | 88(57.52%) | 224(62.57%) |  |
| histological_type | endometrial | 384(75.15%) | 113(73.86%) | 271(75.7%) | 0.7417 |
| histological_type | Mixed and serous | 127(24.85%) | 40(26.14%) | 87(24.3%) |  |
| grade | G1 & G2 | 91(17.81%) | 26(16.99%) | 65(18.16%) | 0.8505 |
| grade | G3 & G4 | 420(82.19%) | 127(83.01%) | 293(81.84%) |  |
| stage | Stage I & Stage II | 370(72.41%) | 112(73.2%) | 258(72.07%) | 0.8768 |
| stage | Stage III & Stage IV | 141(27.59%) | 41(26.8%) | 100(27.93%) |  |

**Table S2 Primer sequence of genes in qRT-PCR**

|  | Forward sequence | Reverse sequence |
| --- | --- | --- |
| FBN1 | GCGGAAATCAGTGTATTGTCCC | CAGTGTTGTATGGATCTGGAGC |
| HIC1 | GTCGTGCGACAAGAGCTACAA | CGTTGCTGTGCGAACTTGC |
| SFRP4 | CCTGGAACATCACGCGGAT | CGGCTTGATAGGGTCGTGC |
| COL11A1 | ACCCTCGCATTGACCTTCC | TTTGTGCAAAATCCCGTTGTTT |
| ONECUT2 | GGAATCCAAAACCGTGGAGTAA | CTCTTTGCGTTTGCACGCTG |
| HOXB9 | CCATTTCTGGGACGCTTAGCA | TGTAAGGGTGGTAGACGGACG |
| DLX4 | CAGCACCTAAACCAGCGTTTC | GAGCTTCTTATACTTGGAGCGTT |
| MSX1 | ACACAAGACGAACCGTAAGCC | CACATGGGCCGTGTAGAGTC |
| TNF | GAGGCCAAGCCCTGGTATG | CGGGCCGATTGATCTCAGC |
| SIX1 | CTGCCGTCGTTTGGCTTTAC | GCTCTCGTTCTTGTGCAGGT |

**Table S3 Multivariate Cox proportional hazards regression analysis of 10 of 31 prognostic EMT-related genes in EC** **in training set.**

| Genes | HR | Low 95%CI | Up 95%CI | P-value |
| --- | --- | --- | --- | --- |
| FBN1 | 1.107 | 1.032 | 1.188 | 0.004 |
| HIC1 | 0.777 | 0.545 | 1.107 | 0.162 |
| SFRP4 | 0.992 | 0.981 | 1.004 | 0.194 |
| COL11A1 | 1.056 | 1.008 | 1.106 | 0.022 |
| ONECUT2 | 1.203 | 0.986 | 1.468 | 0.068 |
| HOXB9 | 1.006 | 1.000 | 1.012 | 0.062 |
| DLX4 | 1.204 | 1.076 | 1.347 | 0.001 |
| MSX1 | 0.999 | 0.998 | 1.000 | 0.140 |
| TNF | 1.027 | 1.005 | 1.049 | 0.017 |
| SIX1 | 1.040 | 0.997 | 1.085 | 0.072 |

**Table S4 The correlation of ERGs expression and risk score with clinical factors.**

| Genes | age | histological_type | grade | stage |
| --- | --- | --- | --- | --- |
| FBN1 | 3.074(0.002) | 3.296(0.001) | 0.744(0.458) | -0.965(0.336) |
| HIC1 | 3.836(1.567e-04) | 4.067(5.792e-05) | 2.529(0.013) | 1.19(0.235) |
| SFRP4 | 1.925(0.055) | 2.781(0.006) | 3.05(0.003) | 3.215(0.001) |
| COL11A1 | -0.98(0.328) | -1.084(0.280) | -2.292(0.022) | -1.653(0.100) |
| ONECUT2 | -2.081(0.038) | -3.847(1.857e-04) | -6.169(1.522e-09) | -2.267(0.024) |
| HOXB9 | -3.219(0.001) | -2.871(0.005) | -1.918(0.056) | -0.261(0.795) |
| DLX4 | -2.054(0.041) | -4.135(4.752e-05) | -4.563(6.344e-06) | -3.207(0.002) |
| MSX1 | 1.269(0.205) | 9.308(8.345e-19) | 4.868(2.897e-06) | 5.118(5.737e-07) |
| TNF | -1.779(0.076) | -3.254(0.001) | -3.538(4.624e-04) | -1.846(0.067) |
| SIX1 | -1.592(0.112) | -1.872(0.063) | -4.345(2.02e-05) | -1.186(0.237) |
| Risk score | -2.311(0.021) | -2.755(0.006) | -5.322(1.621e-07) | -2.822(0.005) |

**Table S5 27 differentially expressed TFs between EC and normal endometrium.**

| Genes | conMean | treatMean | logFC | P-Value | FDR |
| --- | --- | --- | --- | --- | --- |
| CDX2 | 0.011 | 0.873 | 6.362 | 9.43E-05 | 0.000165299 |
| E2F1 | 1.311 | 13.390 | 3.353 | 2.53E-15 | 1.14E-13 |
| EGR1 | 473.976 | 63.140 | -2.908 | 2.45E-13 | 3.19E-12 |
| ELF5 | 0.013 | 1.549 | 6.904 | 4.66E-11 | 2.70E-10 |
| EZH2 | 0.694 | 6.863 | 3.306 | 5.31E-16 | 9.00E-14 |
| FOXA1 | 0.891 | 6.709 | 2.912 | 1.00E-06 | 2.36E-06 |
| FOXA2 | 3.738 | 16.742 | 2.163 | 7.02E-06 | 1.45E-05 |
| FOXM1 | 0.536 | 11.640 | 4.441 | 9.46E-16 | 9.00E-14 |
| FOXP2 | 1.653 | 0.089 | -4.220 | 6.68E-15 | 2.05E-13 |
| GATA6 | 20.518 | 1.109 | -4.210 | 7.59E-16 | 9.00E-14 |
| H2AFX | 14.827 | 77.136 | 2.379 | 2.67E-15 | 1.14E-13 |
| HOXB13 | 0.034 | 3.040 | 6.470 | 2.49E-09 | 9.60E-09 |
| HOXB7 | 7.981 | 32.681 | 2.034 | 1.57E-08 | 5.14E-08 |
| KLF4 | 26.263 | 5.012 | -2.390 | 4.10E-13 | 4.84E-12 |
| LHX2 | 0.018 | 1.093 | 5.947 | 2.11E-11 | 1.32E-10 |
| LMNB1 | 3.310 | 27.655 | 3.063 | 4.63E-15 | 1.66E-13 |
| MITF | 12.573 | 3.011 | -2.062 | 9.98E-08 | 2.85E-07 |
| MYBL2 | 0.605 | 41.086 | 6.085 | 4.83E-16 | 9.00E-14 |
| NR2F2 | 47.972 | 10.693 | -2.166 | 9.81E-13 | 9.48E-12 |
| NR4A1 | 74.287 | 8.932 | -3.056 | 9.58E-11 | 5.23E-10 |
| PBX3 | 7.866 | 1.446 | -2.444 | 1.46E-13 | 2.15E-12 |
| SALL4 | 0.139 | 0.857 | 2.621 | 2.20E-04 | 3.66E-04 |
| SNAI2 | 17.038 | 3.132 | -2.444 | 7.16E-08 | 2.11E-07 |
| SOX17 | 29.928 | 121.296 | 2.019 | 1.25E-09 | 5.05E-09 |
| SPDEF | 10.887 | 76.854 | 2.820 | 1.13E-06 | 2.64E-06 |
| TCF21 | 3.521 | 0.178 | -4.302 | 6.26E-10 | 2.80E-09 |
| TP63 | 0.109 | 0.491 | 2.175 | 0.007 | 0.009 |
